# Bifactor Item Response Theory Model of Acute Stress Response

**DOI:** 10.1371/journal.pone.0065291

**Published:** 2013-06-07

**Authors:** Yebing Yang, Yunfeng Sun, Ying Zhang, Yuan Jiang, Jingjing Tang, Xia Zhu, Danmin Miao

**Affiliations:** 1 Department of Psychology, Fourth Military Medical University, Xi’an City, Shaanxi Province, PR China; 2 Aviation and Diving Medical Center, Navy General Hospital, Beijing City, PR China; 3 Force Development and Military Political Work Academic Department, National Defense University, Beijing City, PR China; 4 Department of Psychology, No. 309 Hospital of Chinese People’s Liberation Army, Beijing City, PR China; 5 Department of Public Health, Second Leading Cadre of Nanjing Military Command, Nanjing City, Jiangsu Province, PR China; Sapienza University of Rome, Italy

## Abstract

**Background:**

Better understanding of acute stress responses is important for revision of DSM-5. However, the latent structure and relationship between different aspects of acute stress responses haven’t been clarified comprehensively. Bifactor item response model may help resolve this problem.

**Objective:**

The purpose of this study is to develop a statistical model of acute stress responses, based on data from earthquake rescuers using Acute Stress Response Scale (ASRS). Through this model, we could better understand acute stress responses comprehensively, and provide preliminary information for computerized adaptive testing of stress responses.

**Methods:**

Acute stress responses of earthquake rescuers were evaluated using ASRS, and state/trait anxiety were assessed using State-trait Anxiety Inventory (STAI). A hierarchical item response model (bifactor model) was used to analyze the data. Additionally, we tested this hierarchical model with model fit comparisons with one-dimensional and five-dimensional models. The correlations among acute stress responses and state/trait anxiety were compared, based on both the five-dimensional and bifactor models.

**Results:**

Model fit comparisons showed bifactor model fit the data best. Item loadings on general and specific factors varied greatly between different aspects of stress responses. Many symptoms (40%) of physiological responses had positive loadings on general factor, and negative loadings on specific factor of physiological responses, while other stress responses had positive loadings on both general and specific factors. After extracting general factor of stress responses using bifactor analysis, significant positive correlations between physiological responses and state/trait anxiety (r = 0.185/0.112, p<0.01) changed into negative ones (r = −0.177/−0.38, p<0.01).

**Conclusion:**

Our results demonstrated bifactor structure of acute stress responses, and positive and negative correlations between physiological responses and stress responses suggested physiological responses could have negative feedback on severity of stress responses. This finding has not been convincingly demonstrated in previous research.

## Introduction

As the number of traumatic events, disasters both natural and manmade, has increased in recent years, acute stress responses (ASR) to these events have become an important research topic in disaster medicine [1], [2]. The possibility that findings may have substantial implications for predicting post-traumatic stress disorder (PTSD) has resulted in a greater push for overall analysis of ASR [3], [4]. Research has provided significant evidence that some of these responses, such as elevated heart rate [5] and cortisol level [6] in acute conditions, can predict PTSD to some extent. However, people affected by traumatic events can demonstrate quite different acute responses. Thus, it is essential for our understanding of the link between ASR and PTSD to first develop a more comprehensive view of the stress response itself.

ASR are complicated outcomes, which will be manifested by most people affected by sudden events. And the developments of ASR of different people varied greatly. Common responses can be classified according to four categories: cognitive, emotional, behavioral, and physiological. These responses are unstable and variable; Grinker and Spiegel described them as a “passing parade of every type of psychological and psychosomatic symptom” [7]. However, in accordance with guidelines provided by the *Diagnostic and Statistical Manual of Mental Disorders-IV* (DSM-IV) published by the American Psychiatric Association in 1994 [8], many studies about PTSD prediction have limited the scope stress responses to those associated with the diagnostic category of acute stress disorder (ASD). As evidence has accumulated, it has been found that acute stress disorder, which describes acute stress responses from a pathological point of view, cannot predict PTSD in a satisfactory way [4]. Although there are descriptions of ASD in draft versions of the DSM-5, the proposed revision has changed the definition and descriptions of ASD to some degree [9]. Moreover, the detailed manifestations and the latent structures of ASD still need to be clarified.

Shalev A.Y. noted that, “In most cases these early responses subside without deliberate intervention. In some survivors, however, the early responses do not remit and are followed by prolonged mental disorders.” [10] In this respect, if we can monitor the severity of acute stress responses dynamically after traumatic events, people who are at high risk of developing PTSD could be targeted for early intervention. Considering the variable nature of acute stress responses, for better assessment of acute stress, it is important to elucidate the latent structure of these responses and the relationships between their different aspects. As an important guidebook in psychiatric field, DSM-5 should make clearer of the relationship between ASR and acute stress disorder (ASD) or post traumatic stress disorder (PTSD), and give suggestions about how we evaluate and predict outcomes of victims, especially when there are too many victims to help using limited medical resources.

To better understand the nature and structure of ASR, we have developed a tool to identify the variety and severity of these responses in previous research, which we named the Acute Stress Response Scale (ASRS) [11], consisting of six sub-scales and 112 items. The first four sub-scales are the four types of stress response mentioned above, the fifth sub-scale measures psychiatric symptoms, and the sixth sub-scale measures reduced work efficiency. Psychiatric symptoms, contained in the fifth sub-scale, are fundamentally manifestations of cognition, emotions, behaviors, or physiological responses. After sudden events, some people may display severe symptoms, and may have greater incidence of ASD or PTSD. This sub-scale was compiled by some severe symptoms from other sub-scales, in order to classify individuals with serious responses, who might need special psychiatric treatments. The first five sub-scales include 110 items, reflecting the main stress responses. The two items that pertain to work efficiency have been removed from our analysis in this study, because these two items are different from other items. Firstly, these two items are comprehensive results of different aspects of ASR, not simple symptoms after sudden events like items from other sub-scales. Secondly, compared with other sub-scales, too few items may cause relatively great bias on the estimation of the item parameters. So, we decided to drop these two items from our analysis.

In our earlier study, we found that there were high correlations between these five sub-scales. There are two probable explanations of this phenomenon. The first explanation is that different aspects of stress are highly correlated as individual sub-scales. The second explanation is that the items making up each sub-scale in the original construct are actually multidimensional, reflecting both a general factor for acute stress responses, and specific factors for different aspects of stress responses. Given the first situation, a one-dimension model should have a good fit. Whereas if the second situation were the case, an item level multidimensional model would be more appropriate.

With the development of item response theory (IRT), multidimensional models have been used much more frequently in the factor analysis of psychological scales [12], [13]. This type of modeling is called bifactor analysis, which will be explained in the Methods and Results section of this paper. Such general and specific factor structures have been shown to exist in many psychological and psychiatric symptoms including the relationship between depression and anxiety [14], and cognitive impairment in schizophrenia [15]. This can also be seen in the structure of psychiatric diagnostic screening questionnaires (PDSQ) [16].

These types of models have the advantage of analyzing data systematically and understanding related variables comprehensively. If applied in the assessment of stress responses they will also be helpful in examining the structure of the different aspects of the stress response. Additionally, IRT lends itself naturally to the use of computerized adaptive testing (CAT) which would allow the severity of stress responses to be monitored dynamically with efficiency and accuracy [17].

The present study tested the hypothesis that every item of the Acute Stress Response Scale was influenced by both a general factor (acute stress) and one of five specific factors (cognitive changes, emotional responses, behavioral changes, physiological responses, and psychiatric symptoms). We used bifactor analysis to model the ASRS according to the hypothesized latent factor structure. In order to support this hypothesis, we also conducted a model fit comparison between one-dimensional,five-dimensional, and bifactor models. Additionally, we analyzed correlations between state/trait anxiety and acute stress responses (calculated from five-dimensional and bifactor models) to demonstrate the advantages of bifactor analysis. Through these analyses we hope to provide useful data and findings for the revision of the DSM-5, as well as better intervention of acute stress responses. If we could found a general factor of different aspects of ASR, it might serve as an indicator for selecting potential patients of ASD and PTSD. Additionally, there also is a sub-scale, named as “psychiatric symptoms”, which also may provide some information for predicting occurrence of ASD and PTSD. However, the relationship between general factor of ASR or severity of “psychiatric symptoms” and incidences of ASD and PTSD should be studied in the future with longitudinal design. And computerized adaptive testing of ASR based on bifactor item response theory analysis may have benefits for these kinds of studies.

## Methods

### Ethics Statement

The Ethics Committee of the Fourth Military Medical University approved this study. All the participants gave written informed consent and agreed to complete the investigation. And all the participants were adults.

### Participants and Procedures

Participants in the study were male rescuer workers from Guizhou Province, Sichuan Province and Shaanxi Province that participated in rescuing activities after the great earthquake in 2008, in Sisun, China (N = 983). They mainly worked searching for victims and cleaning-up areas affected by the earthquake. Of the participants, 843 returned valid questionnaires (mean age 19.9 years, SD = 2.3). There are some differences between the standards of “adult” between China and United States. In China, people older than 16 years old could be called “adult”, and they should be responsible for their conducts. The youngest subject in our study was 17 years old, and the oldest was 24 years old. So subjects enrolled in our study could attend our investigation without their parents’ permissions. All the participants were male. The selection and exclusion criteria for including participants in the study were as follows.

#### Selection criteria

Being capable of understanding and speaking Chinese; ability to understand the questionnaires and being capable of independently completing the test; returning valid answers for all the investigating items. Not suffering from organic brain damage or infections, and not having been treated for a mental illness.

#### Exclusion criteria

Not being capable of understanding Chinese or the test content; suffering from organic brain damage or infection; Inability complete the investigations due to physical illness.

### Measures

#### Acute Stress Response Scale (ASRS) [11]

The authors in an earlier study had developed the ASRS. The data used in the present study are part of the reliability and validity studies conducted on the ASRS. However, in order to make sure the accuracy of this study, we have used more rigorous standard when selecting valid data. We have chosen data without any missing values, while we chose data with less than 5 missing values as valid data in our previous study. ASRS was developed on the basis of previous literature on stress research and through our practice of doing rescue work during earthquakes. The ASRS is designed to assess the main patterns of stress manifestation. The scale consists of 112 items in six sub-scales: cognitive changes, emotional responses, behavioral changes, physiological responses, psychiatric symptoms, and reduced work efficiency. Reduced work efficiency, which is assessed by two items, was used in the initial evaluation on reduction of work efficiency and therefore, data from the other five sub-scales, with the exception of reduced work efficiency was used in the current study. “Yes” or “No” responses were made to the remaining 110 items. In our earlier study the acceptable reliability and validity of this scale was demonstrated. All sub-scales used in the present study had acceptable α coefficients: Cognitive changes α = 0.89, emotional responses α = 0.89, behavioral changes α = 0.84, physiological responses α = 0.88 and psychiatric symptoms α = 0.74. The total scale has α = 0.85. This scale was developed using Chinese language.

#### State-Trait Anxiety Inventory (STAI) [18]

STAI is a self-report scale consisting of 40 items. In the scale, the first 20 items are mainly used to evaluate the degree of participants’ recent anxiety (state anxiety) and the last 20 items are used to assess the participants’ usual anxiety level (trait anxiety). Participants respond to all items using a scale of 1–4, such that in the first 20 items the current level of anxiety is measured as follows: 1 (*None*), 2 (*Some*), 3 (*Moderate*) and 4 (*Very obviously*), whereas in the last 20 items the usual level of anxiety is measured as follows: 1 (*Almost never*), 2 (*Sometimes*), 3 (*Often*) and 4 (*Always*). This scale had been translated and validated using Chinese sample.

### Data Analysis

**Figure 1:**
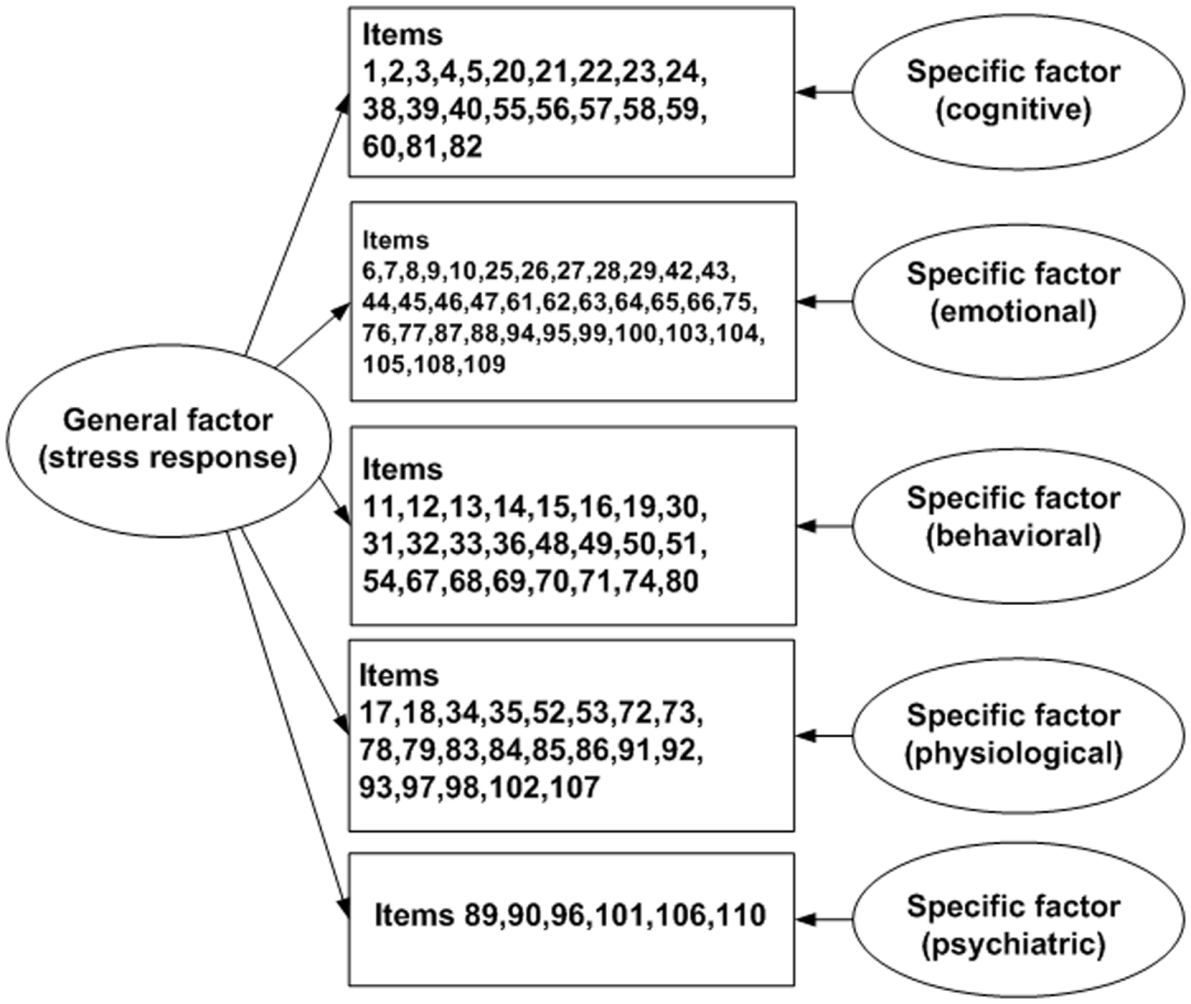
Bifactor structure of ASRS. Structure of the hierarchical model of the ASRS constructed using bifactor analysis. In the original scale, 110 items load on five subscales respectively. In the bifactor analysis, all the items have loadings on both the general factor and one of the subscale specific factors.

First, we conducted classical testing theory analyses of ASRS, including descriptive analyses of the sub-scale scores (the combined results of scores of the corresponding items), and correlation analysis between items and the sub-scale scores, as well as the total score of all items. Second, in order to prove the advantage of bifactor model analysis, we conducted several confirmative factor analyses to compare the model fit of different models, including one-dimensional, five-dimensional, and bifactor models. The five-dimensional model was in accordance with the original construct of the scale, while in the bifactor model, every item has two factor loadings, one for a general factor, and another for one of the specific factors. It was a hierarchical model could be seen as the combination of the one-dimensional and five-dimensional models (see Figure 1). Correlations were calculated between each of the five sub-scale scores obtained via five-dimensional model analysis and state/trait anxiety scores. In addition, the general and specific factor scores obtained via a bifactor analysis were also correlated with state/trait anxiety. Through bifactor analysis, we could gain a more comprehensive knowledge of the reliability of the scale, and correlations between general and specific factor scores. Correlations between general and specific factors and other psychological variables could be seen as representing evidence of the validity of the scale under the hierarchical bifactor analysis.

Descriptive and correlation analyses were conducted using SPSS for Windows 13.0. Model fit comparison was conducted based on confirmative factor analyses completed using Lisrel 8.50. Bifactor analysis was conducted using program POLYBIF [12]. POLYBIF is designed for analysis of bifactor models, with the merit of easy operation and trustworthy results. The program can analyze bifactor models as a multidimensional IRT models, and it results in collecting the loading scores of the general factor and specific factors under each item and calculating the test information curve for the general factor.

## Results

### CTT Analysis of ASRS

We conducted descriptive analysis for the five sub-scales and all 110 items of ASRS. And the endorsing rate for the items varied from 8.4% to 36.5%. Correlation analysis revealed that all the five sub-scales had moderate significant positive correlations with each other (see Table 1). Nearly all the 110 items had low to moderate significant positive correlations with all five sub-scales and total scores (see Table 2). The only exception was Item 4, which had no significant correlation with physiological responses. These results suggested that high correlations between different sub-scales might be because of multidimensionality on the level of the items. Thus a multidimensional model, such as a hierarchical bifactor model, should be used to analyze this scale.

**Table 1:** Correlations between Different Sub-scales of ASRS.

	Cognitive	Emotional	Behavioral	Physiological	Psychiatric
Cognitive	1				
Emotional	0.790**	1			
Behavioral	0.752**	0.779**	1		
Physiological	0.675**	0.714**	0.664**	1	
Psychiatric	0.506**	0.596**	0.625**	0.493**	1

** p<0.01.

Cognitive is short for “Cognitive changes”; emotional is short for “Emotional responses”; behavioral is short for “Behavioral changes”; physiological is short for “Physiological responses”; and psychiatric is short for “Psychiatric symptoms.”

**Table 2:** Correlations between Items and ASRS Scales.

Item	Sub-scale	Cognitive	Emotional	Behavioral	Physiological	Psychiatric	Total
Item1	Cognitive	0.467	0.349	0.318	0.170	0.233	0.370
Item2	Cognitive	0.452	0.341	0.366	0.204	0.305	0.386
Item3	Cognitive	0.427	0.298	0.303	0.142	0.183	0.329
Item4	Cognitive	0.353	0.268	0.305	0.026	0.266	0.276
Item5	Cognitive	0.477	0.331	0.327	0.125	0.229	0.357
Item6	Emotional	0.312	0.391	0.299	0.228	0.127	0.346
Item7	Emotional	0.400	0.510	0.402	0.379	0.208	0.475
Item8	Emotional	0.406	0.510	0.369	0.350	0.233	0.464
Item9	Emotional	0.330	0.477	0.385	0.266	0.318	0.420
Item10	Emotional	0.385	0.446	0.346	0.157	0.287	0.387
Item11	Behavioral	0.357	0.382	0.505	0.335	0.210	0.433
Item12	Behavioral	0.321	0.277	0.442	0.110	0.233	0.320
Item13	Behavioral	0.245	0.277	0.398	0.172	0.231	0.304
Item14	Behavioral	0.368	0.408	0.489	0.216	0.276	0.416
Item15	Behavioral	0.410	0.394	0.511	0.344	0.178	0.453
Item16	Behavioral	0.403	0.414	0.478	0.534	0.181	0.496
Item17	Physiological	0.277	0.248	0.267	0.452	0.070	0.332
Item18	Physiological	0.355	0.380	0.403	0.380	0.337	0.426
Item19	Behavioral	0.395	0.372	0.496	0.228	0.361	0.420
Item20	Cognitive	0.532	0.482	0.453	0.255	0.326	0.489
Item21	Cognitive	0.528	0.413	0.460	0.288	0.329	0.474
Item22	Cognitive	0.545	0.378	0.411	0.305	0.224	0.454
Item23	Cognitive	0.449	0.292	0.347	0.174	0.256	0.354
Item24	Cognitive	0.478	0.352	0.291	0.399	0.144	0.418
Item25	Emotional	0.358	0.432	0.372	0.342	0.235	0.423
Item26	Emotional	0.340	0.367	0.266	0.330	0.115	0.362
Item27	Emotional	0.325	0.486	0.379	0.231	0.297	0.411
Item28	Emotional	0.340	0.429	0.354	0.279	0.302	0.402
Item29	Emotional	0.341	0.487	0.368	0.301	0.285	0.429
Item30	Behavioral	0.360	0.349	0.441	0.453	0.154	0.433
Item31	Behavioral	0.393	0.354	0.428	0.533	0.121	0.458
Item32	Behavioral	0.362	0.433	0.559	0.290	0.377	0.462
Item33	Behavioral	0.348	0.353	0.519	0.307	0.324	0.423
Item34	Physiological	0.480	0.468	0.451	0.570	0.278	0.543
Item35	Physiological	0.403	0.417	0.365	0.625	0.197	0.495
Item36	Behavioral	0.422	0.353	0.495	0.323	0.292	0.439
Item37	Cognitive	0.465	0.442	0.388	0.582	0.177	0.513
Item38	Cognitive	0.589	0.445	0.420	0.463	0.232	0.529
Item39	Cognitive	0.546	0.437	0.362	0.415	0.194	0.488
Item40	Cognitive	0.527	0.447	0.419	0.251	0.332	0.467
Item41	Cognitive	0.408	0.435	0.446	0.319	0.436	0.460
Item42	Emotional	0.424	0.462	0.380	0.388	0.269	0.466
Item43	Emotional	0.408	0.448	0.440	0.278	0.340	0.447
Item44	Emotional	0.408	0.536	0.433	0.250	0.405	0.473
Item45	Emotional	0.422	0.537	0.410	0.318	0.411	0.489
Item46	Emotional	0.388	0.499	0.397	0.324	0.345	0.461
Item47	Emotional	0.365	0.453	0.284	0.329	0.131	0.403
Item48	Behavioral	0.357	0.348	0.502	0.294	0.359	0.419
Item49	Behavioral	0.362	0.442	0.511	0.229	0.421	0.442
Item50	Behavioral	0.332	0.420	0.516	0.296	0.435	0.444
Item51	Behavioral	0.301	0.346	0.412	0.288	0.384	0.383
Item52	Physiological	0.382	0.412	0.485	0.415	0.423	0.476
Item53	Physiological	0.351	0.343	0.279	0.609	0.164	0.430
Item54	Behavioral	0.290	0.271	0.416	0.160	0.264	0.318
Item55	Cognitive	0.569	0.462	0.408	0.541	0.311	0.550
Item56	Cognitive	0.477	0.341	0.328	0.406	0.284	0.432
Item57	Cognitive	0.457	0.428	0.474	0.309	0.376	0.471
Item58	Cognitive	0.428	0.306	0.227	0.349	0.116	0.361
Item59	Cognitive	0.534	0.458	0.373	0.481	0.223	0.512
Item60	Cognitive	0.541	0.455	0.394	0.489	0.229	0.520
Item61	Emotional	0.380	0.466	0.355	0.314	0.228	0.429
Item62	Emotional	0.383	0.490	0.401	0.402	0.321	0.475
Item63	Emotional	0.378	0.481	0.430	0.276	0.374	0.450
Item64	Emotional	0.300	0.429	0.380	0.258	0.425	0.398
Item65	Emotional	0.451	0.555	0.420	0.341	0.329	0.506
Item66	Emotional	0.446	0.541	0.374	0.452	0.280	0.513
Item67	Behavioral	0.336	0.392	0.488	0.304	0.494	0.435
Item68	Behavioral	0.355	0.378	0.552	0.334	0.375	0.450
Item69	Behavioral	0.342	0.462	0.491	0.303	0.406	0.456
Item70	Behavioral	0.382	0.482	0.538	0.314	0.393	0.486
Item71	Behavioral	0.321	0.403	0.426	0.353	0.494	0.432
Item72	Physiological	0.351	0.380	0.294	0.611	0.226	0.451
Item73	Physiological	0.354	0.357	0.295	0.630	0.151	0.444
Item74	Behavioral	0.375	0.417	0.519	0.345	0.521	0.472
Item75	Emotional	0.341	0.425	0.349	0.441	0.240	0.435
Item76	Emotional	0.378	0.482	0.434	0.237	0.404	0.443
Item77	Emotional	0.386	0.479	0.396	0.340	0.325	0.456
Item78	Physiological	0.390	0.428	0.356	0.644	0.205	0.498
Item79	Physiological	0.444	0.453	0.391	0.677	0.234	0.539
Item80	Behavioral	0.440	0.378	0.445	0.449	0.250	0.470
Item81	Cognitive	0.508	0.422	0.432	0.397	0.292	0.490
Item82	Cognitive	0.526	0.423	0.364	0.541	0.202	0.509
Item83	Physiological	0.298	0.334	0.356	0.494	0.341	0.413
Item84	Physiological	0.251	0.355	0.389	0.349	0.476	0.387
Item85	Physiological	0.272	0.244	0.211	0.536	0.130	0.341
Item86	Physiological	0.335	0.336	0.372	0.494	0.314	0.425
Item87	Emotional	0.313	0.434	0.382	0.275	0.353	0.404
Item88	Emotional	0.394	0.529	0.427	0.353	0.444	0.492
Item89	Psychiatric	0.392	0.427	0.472	0.341	0.713	0.479
Item90	Psychiatric	0.335	0.427	0.455	0.370	0.733	0.468
Item91	Physiological	0.405	0.441	0.471	0.388	0.515	0.487
Item92	Physiological	0.405	0.424	0.347	0.625	0.251	0.496
Item93	Physiological	0.356	0.362	0.388	0.490	0.395	0.447
Item94	Emotional	0.392	0.497	0.334	0.548	0.238	0.496
Item95	Emotional	0.398	0.514	0.438	0.301	0.366	0.474
Item96	Psychiatric	0.408	0.440	0.512	0.348	0.698	0.498
Item97	Physiological	0.392	0.471	0.503	0.423	0.474	0.508
Item98	Physiological	0.431	0.461	0.352	0.606	0.202	0.510
Item99	Emotional	0.278	0.399	0.314	0.191	0.275	0.343
Item100	Emotional	0.343	0.497	0.376	0.293	0.457	0.442
Item101	Psychiatric	0.343	0.426	0.429	0.338	0.679	0.453
Item102	Physiological	0.366	0.421	0.358	0.567	0.238	0.473
Item103	Emotional	0.443	0.511	0.381	0.540	0.280	0.525
Item104	Emotional	0.378	0.452	0.304	0.365	0.241	0.426
Item105	Emotional	0.368	0.465	0.348	0.440	0.206	0.454
Item106	Psychiatric	0.295	0.421	0.381	0.293	0.596	0.413
Item107	Physiological	0.311	0.439	0.426	0.377	0.502	0.448
Item108	Emotional	0.379	0.519	0.331	0.469	0.266	0.481
Item109	Emotional	0.331	0.458	0.381	0.327	0.241	0.423
Item110	Psychiatric	0.274	0.293	0.294	0.309	0.629	0.349

Cognitive is short for “Cognitive changes”; emotional is short for “Emotional responses”; behavioral is short for “Behavioral changes”; physiological is short for “Physiological responses”; and psychiatric is short for “Psychiatric symptoms.”

### Model Comparison

In order to provide evidence for the conclusion above, we compared model fit between three competing models: one-dimensional, five-dimensional, and bifactor. The model constructs are explained above. As shown in Table 3, the bifactor model demonstrated the best model fit indices. Also, we can see that changes of the chi-squares are all significant when we compare the other two models with the hierarchical bifactor model.

**Table 3:** Model comparison between unidimensional, five-dimensional and bifactor models.

	RMSEA	χ^2^	Df	△χ^2^	△df
Unidimensional	0.062	25157.35	5885	13561.16*	116
Five-dimensional	0.060	23801.48	5884	12295.29*	115
Bifactor	0.034	11506.19	5769	−	−

* p<0.05;

△χ^2^ and △df represent model fit comparison between unidimensional, five-dimensional, and bifactor models.

### Bifactor Analyses of the ASRS

**Figure 2:**
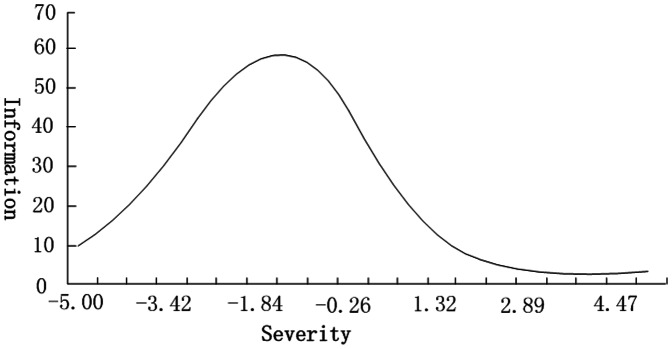
Test information of ASRS. The test information curve of the ASRS based on the bifactor analysis for the general stress factor. X-axis represents severity of the general factor (severity), which had been standardized (0 being average, 1 being a standard deviation). The Y-axis represents the test information value. Test information is a type of reliability criterion in IRT models, the larger the test information value, the less the measurement error and the better the reliability. In contrast to models constructed using CTT, in IRT models, there is a test information value corresponding to every severity point, representing the reliability at that level of severity. We obtained the test information curve by connecting all these values.

As an IRT model, the bifactor model has many parameters. *Slope* is a measure of an item’s ability to discriminate between different severities of the trait being measured. *Location* is a measure of the extent to which responses to an item indicate greater or lesser severity of the trait being measured. Finally, *severity* measures severity of a participant’s symptom. *Slop* and *location* are item parameters, while *severity* is a participant parameter. Also, in the IRT model, reliability or measurement error is reflected by item and test *information*, the more accurate the measurement, the greater the information gained. Unlike classical testing theory (CTT) in which the reliability of a scale is just one value, item or test information is a mathematical function in which trait severity is on the X-axis and the information value is on the Y-axis. In this way we can determine at what degree of severity the scale will give results with the highest accuracy. As can be seen in Figure 2, severity is a standardized score (0 being average and 1 being a standard deviation). *Factor loading* has the same meaning with loadings in other results of factor analyses.

In this study we found that most items have moderate to large loadings on the general factor; 86 items had greater loadings on the general factor than on specific factors, whereas the other 24 items had greater loadings on specific factors than on the general factor. That is, most items reflected the severity of a general factor (i.e. stress response) more than that of the specific factors. However, many items also had moderate to large loadings on specific factors.

Interestingly, there were some items that had positive loadings on the general factor, but negative loadings on specific factors. This means that independent of the general factor, the item was negatively correlated with the specific factor it was originally intended to measure. This was especially salient for items grouped in the sub-scale “physiological responses”; more than 40% of items had positive loadings on general factor, and negative loadings on the specific factor of physiological responses.

As for the scores of general and specific factors, correlation analysis found notable results (see Table 4). We found that all five sub-scales had significant positive moderate to large correlations with the general factor. Most of the sub-scales had significant low to moderate correlations with the five specific factors. However, all five sub-scales, except physiological responses, had significant negative correlations with the specific factor of physiological responses, and psychiatric symptoms had a large correlation with the specific factor of psychiatric symptoms (r = 0.788, p<0.01). These results suggested the correlations between different aspects of the stress response are complicated and need to be studied comprehensively. Test information showed that ASRS could accurately measure a general factor between (−5, 1.8), but was less accurate for factors larger than 2, which is to say, this scale could discriminate slight to moderate stress responses, but could not discriminate severe stress responses very well (Figure 2). Additionally, cognitive changes had the least mean severity location, and psychiatric symptoms had the largest mean severity location (see Table 5).

**Table 4:** Correlations between CTT and Bifactor Analysis Results of ASRS.

	Cognitive	Emotional	Behavioral	Physiological	Psychiatric
General factor	0.805**	0.795**	0.733**	0.885**	0.453**
Specific factor of Cognitive	0.526**	0.264**	0.319**	−0.099**	0.259**
Specific factor of Emotional	0.246**	0.501**	0.308**	−0.073*	0.300**
Specific factor of Behavioral	0.235**	0.277**	0.560**	−0.054	0.383**
Specific factor of Physiological	−0.174**	−0.212**	−0.297**	0.127*	−0.352**
Specific factor of Psychiatric	0.126**	0.179**	0.256**	0.030	0.788**

* p<0.05;

** p<0.01.

Cognitive is short for “Cognitive changes”; emotional is short for “Emotional responses”; behavioral is short for “Behavioral changes”; physiological is short for “Physiological responses”; and psychiatric is short for “Psychiatric symptoms.”

**Table 5:** Main results of bifactor analysis (M±SD).

	Number of items	Locations	Loadings on the general factor	Loadings on the specific factors
Cognitive	23	0.39±0.26	0.58±0.19	0.32±0.22
Emotional	36	0.69±0.30	0.62±0.10	0.32±0.20
Behavioral	24	0.74±0.38	0.56±0.13	0.41±0.19
Physiological	21	0.52±0.42	0.72±0.09	0.27±0.15
Psychiatric	6	1.14±0.99	0.70±0.06	0.52±0.07

Cognitive is short for “Cognitive changes”; emotional is short for “Emotional responses”; behavioral is short for “Behavioral changes”; physiological is short for “Physiological responses”; and psychiatric is short for “Psychiatric symptoms.”

To compare the influence of the different analyses on the assessment results and analyze the latent construct of acute stress responses, we used both the five-dimensional model and the bifactor model to calculate the correlations between the stress response and state/trait anxiety. Five-dimensional model analysis showed that there were low to moderate significant positive correlations between all five sub-scales and state/trait anxiety. Under bifactor model analysis, both state and trait anxiety had low significant positive correlations with the general factor. And state anxiety also had low significant positive correlations with specific factors of cognitive changes, emotional responses, behavioral changes and psychiatric symptoms, and had low significant negative correlation with specific factor of physiological responses. Trait anxiety had significant moderate positive correlations with the specific factors of cognitive changes, emotional responses and behavioral changes, and had significant low positive correlation with the specific factor of psychiatric symptoms, but significant low negative correlation with the specific factor of physiological responses.

Compared with the five-dimensional model analysis, all correlations between state anxiety and the specific factors of stress responses had decreased to some extent, especially for the correlation with the specific factor of physiological responses, in which the positive correlation (r = 0.185, p<0.01) changed into a negative one (r = −0.177, p<0.01). Correlations between trait anxiety and four of the specific factors of stress responses did not show obvious changes, whereas correlation with the specific factor of physiological responses changed from a significant positive one (r = 0.112, p<0.01) to a significant negative one (r = −0.38, p<0.01) (see Table 6).

**Table 6:** Correlations between State/trait Anxiety and ASRS Results Under CTT and Bifactor Analysis.

	State Anxiety	Trait Anxiety
Cognitive	0.338**	0.418**
Emotional	0.334**	0.407**
Behavioral	0.334**	0.394**
Physiological	0.185**	0.112**
Psychiatric	0.223**	0.369**
General factor	0.285**	0.226**
Specific factor of Cognitive	0.270**	0.504**
Specific factor of Emotional	0.181**	0.426**
Specific factor of Behavioral	0.197**	0.399**
Specific factor of Physiological	−0.177**	−0.380**
Specific factor of Psychiatric	0.097**	0.258**

* p<0.05;

** p<0.01.

Cognitive is short for “Cognitive changes”; emotional is short for “Emotional responses”; behavioral is short for “Behavioral changes”; physiological is short for “Physiological responses”; and psychiatric is short for “Psychiatric symptoms.”

## Discussion

The results of this study clarify the latent dimensionality of acute stress responses from a comprehensive physiological view. On the one hand, we treated acute stress responses as a normal physiological process. And on the other had, we also considered potential patients of ASD and PTSD, who may demonstrate severe symptoms in the early stage of coping with stress. Additionally, a hierarchical model based on IRT-based bifactor analysis permitted us to study two kinds of relationships. The first is between the general factor of stress responses and specific factors of different aspects of stress responses. The second is between general/specific factors of stress responses and other related psychological variables, such as state/trait anxiety. Our results, which were based on data of rescue workers responding to earthquake victims, supported the bifactor structure of acute stress responses, and provided important insights into the components of the responses, which may help with further revision of the DSM-5. Additionally, our results have provided more evidence showing the advantages of IRT analysis in clinical assessment [19].

### Relationship between General Factor and Specific Factors of the Stress Response

Through bifactor modeling of self-reported symptoms on the ASRS, we found support for the existence of general and specific factor structures of stress responses. However, the relative influence of the general factor, compared with the specific factors, varied with the aspects of responses. Symptom clusters such as hypoprosexia and nightmares in cognitive changes, and anxiety in emotional responses, yielded relatively high general components and small specific components. In contrast, symptom clusters such as disorientation in cognitive changes and “being isolated from others” in behavioral changes, yielded relatively small general components and high specific components. Such findings are of clinical interest because symptoms that had higher specific components and lower general components may need specially targeted treatments, while those with higher general components might benefit most from interventions that reduce overall stress levels.

Interestingly, some symptoms of physiological responses yielded large general components and small to moderate negative specific components. Analyzed from the point of view of psychodynamics and stress theory, stress can be seen as a kind of energy, reflected by all kinds of responses. Physiological responses can reflect the severity of stress on the one hand, and they can also release some energy to decrease the severity of stress on the other hand, being similar with some kind of negative feedback in neural network. That is to say, physiological responses should have both positive and negative correlations with stress severity, just like the correlations we have found between anxiety and depression [14]. But this kind of relationship cannot be made clear based on CTT analysis. With the help of bifactor model analysis, we were able to obtain evidence of this complicated relationship for the first time. The positive correlation could be reflected by the positive loadings on the general factor (stress responses), whereas the negative correlation could be seen from the negative loadings on specific factor of physiological responses.

With the help of bifactor analysis, we were also able to obtain severity parameters for every item on the general factor of stress responses. These results suggested that in terms of sub-scales, these responses ranged from slightest to most severe in the following order: cognitive changes, physiological responses, emotional responses, behavioral changes, psychiatric symptoms. It is possible that this order also reflects the stages of development for acute stress responses, which will be studied in continuing research. If so, it could provide valuable information for understanding ASR. On the item level, we could see that “meaningless life” was the most severe symptom, and “elevated heart rate” was the slightest manifestation.

Moreover, examining item location parameters also can help us to better understand the function of psychological scales. As for ASRS, we found that the largest item severity location was 1.73, and the smallest was 0.048, showing that these items could assess slight to moderate severe stress responses well, but were less accurate for very severe responses. This may be because most of the items were developed from a normal physiological view. More pathological stress responses, such as symptoms described for ASD in the DSM-5 proposed revision, should be added in order to improve the function of this scale.

### Relationship between the Stress Responses and other Related Psychological Variables

If we were able to gain more comprehensive knowledge of the inner structure of stress responses, we would also be able to gather more details about the relationship between stress responses and other psychological variables, such as state/trait anxiety. In this study, for the first time we found evidence that physiological responses have a complicated relationship with state/trait anxiety. The correlations between physiological responses and state/trait anxiety changed from significantly positive ones to significant negative ones when the effects of a general factor were excluded. For other aspects of stress responses, both a general factor and specific factors have significant positive correlations with state/trait anxiety. Comparing the values of these correlations, we could see that correlations between stress responses under CTT analysis could be explained by the combination of correlations between general and specific factors of stress responses with state/trait anxiety. However, this is also one of the drawbacks of this study. We hadn’t included enough validity indices, and couldn’t draw the big picture of relationship between stress responses and other related psychological variables, such as resilience factors. And it could be more informative if we could understand the role of ASR in the dynamic psychological developments for traumatic stress victims, which should be made clear in the future.

### Implication for DSM-5

According to review of Bryant RA et al. [20], the DSM-5 may treat ASD as severe manifestation of acute stress responses without requiring specific clusters, and this is valuable for improving diagnosis. However, manifesting acute stress responses is one type of normal process after traumatic experiences. We suggest that it should be described from a comprehensive perspective. Descriptions and assessment of ASR from a normal physiological view should be emphasized. And ASD can be defined as severe stress responses. The results of our study showed that different responses had varied loadings on the general factor (stress responses) and the corresponding severity level also varied greatly. Therefore using a generated score of the general factor, as well as a score of psychiatric symptoms based on the bifactor model may give a more accurate evaluation of stress severity than an added score of items. On the other hand, a computerized adaptive testing system based on bifactor item response theory analysis could be developed, with which severity of ASR could be checked dynamically, and potential patients of ASD and PTSD could be distinguished early.

This study is a continuation of our earlier research and still represents preliminary results for future studies. This research had a number of obvious limitations. First, longitudinal data is missing, and we could not obtain the predictive power of ASR for PTSD. Second, the results were mainly based on self-reported data, which would be more reliable if cognitive experiments had been conducted. Third, we have only explored the structure of stress responses, and a computerized adaptive testing system for ASR needs to be developed in the future. Fourth, the sample used in this study was entirely male, and its generalizability should be studied in the future.
